# Characterization of a Disordered above Room Temperature Skyrmion Material Co_8_Zn_8_Mn_4_

**DOI:** 10.3390/ma14164689

**Published:** 2021-08-19

**Authors:** Melissa E. Henderson, James Beare, Sudarshan Sharma, Markus Bleuel, Pat Clancy, David G. Cory, Michael G. Huber, Casey A. Marjerrison, Mathew Pula, Dusan Sarenac, Evan M. Smith, Kirill Zhernenkov, Graeme M. Luke, Dmitry A. Pushin

**Affiliations:** 1 Institute for Quantum Computing, University of Waterloo, Waterloo, ON N2L 3G1, Canada; dcory@uwaterloo.ca (D.G.C.); dsarenac@uwaterloo.ca (D.S.); K.zhernenkov@fz-juelich.de (K.Z.); dmitry.pushin@uwaterloo.ca (D.A.P.); 2 Department of Physics & Astronomy, University of Waterloo, Waterloo, ON N2L 3G1, Canada; 3 Department of Physics and Astronomy, McMaster University, Hamilton, ON L8S 4M1, Canada; bearej@mcmaster.ca (J.B.); shars64@mcmaster.ca (S.S.); pulam@mcmaster.ca (M.P.); smithem4@mcmaster.ca (E.M.S.); luke@mcmaster.ca (G.M.L.); 4 National Institute of Standards and Technology, Gaithersburg, MD 20899, USA; markus.bleuel@nist.gov (M.B.); michael.huber@nist.gov (M.G.H.); 5 Department of Materials Science and Engineering, University of Maryland, College Park, MD 20742, USA; 6 Brockhouse Institute for Materials Research, Hamilton, ON L8S 4M1, Canada; clancyp@mcmaster.ca (P.C.); caseymarjerrison@gmail.com (C.A.M.); 7 Department of Chemistry, University of Waterloo, Waterloo, ON N2L 3G1, Canada; 8 Jülich Centre for Neutron Science at Heinz Maier-Leibnitz Zentrum, Forschungszentrum Jülich GmbH, 85748 Garching, Germany

**Keywords:** skyrmion, magnetism, disordered

## Abstract

Topologically nontrivial spin textures host great promise for future spintronic applications. Skyrmions in particular are of burgeoning interest owing to their nanometric size, topological protection, and high mobility via ultra-low current densities. It has been previously reported through magnetic susceptibility, microscopy, and scattering techniques that Co${}_{8}$Zn${}_{8}$Mn${}_{4}$ forms an above room temperature triangular skyrmion lattice. Here, we report the synthesis procedure and characterization of a polycrystalline Co${}_{8}$Zn${}_{8}$Mn${}_{4}$ disordered bulk sample. We employ powder X-ray diffraction and backscatter Laue diffraction as characterization tools of the crystallinity of the samples, while magnetic susceptibility and Small Angle Neutron Scattering (SANS) measurements are performed to study the skyrmion phase. Magnetic susceptibility measurements show a dip anomaly in the magnetization curves, which persists over a range of approximately 305 K–315 K. SANS measurements reveal a rotationally disordered polydomain skyrmion lattice. Applying a symmetry-breaking magnetic field sequence, we were able to orient and order the previously jammed state to yield the prototypical hexagonal diffraction patterns with secondary diffraction rings. This emergence of the skyrmion order serves as a unique demonstration of the fundamental interplay of structural disorder and anisotropy in stabilizing the thermal equilibrium phase.

## 1. Introduction

Topologically protected states are ubiquitous in nature, appearing in disparate physical systems spanning condensed matter phases, such as liquid crystals [1], to cosmological strings [2]. Magnetic skyrmions represent a particularly exciting class of these topological objects, commonly stabilized by the antisymmetric Dzyaloshinskii–Moriya (DM) exchange interaction in noncentrosymmetric bulk materials [3,4,5,6]. Manifesting as quasiparticle nanoscale magnetic spin configurations with a whirling vortex-like structure in magnetic textures, their integer topological charges equate to a nontrivial mapping of the magnetization from real space to the order parameter space of the two-dimensional unit sphere [7]. This countable “winding” property gives rise to Berry curvatures, which can be expressed in terms of the emergent electric and magnetic fields, wherein each skyrmion tube corresponds to one quantum of the emergent magnetic flux [8]. From this combination of the quantized topology of skyrmions—both in real magnetization fields and emergent fields—and their solitary particle-like behaviour, arises novel phenomena such as a topological Hall effect [9,10], multiferroic behaviour [11,12,13], and current-driven dynamics five to six orders of magnitude smaller than those currently required to drive domain walls in ferromagnets [8,14,15]. Ultimately, their electric controllability, in combination with their nanometric size, make magnetic skyrmions prime candidates for potential information carriers in quantum information science [16,17,18,19,20].

Magnetic skyrmions are known to occur in materials lacking inversion symmetry, owing to chiral crystal structures in bulk magnets [2,4,10,21,22,23] and thin films [3,24,25,26], or nonequivalent interfaces in multilayers and ultra-thin films [27,28,29,30]. In noncentrosymmetric chiral lattices, the competition of the Heisenberg exchange interaction with the antisymmetric DM exchange interaction [31,32] tends to stabilize helical ground states in ferromagnetic crystals [10]. The application of a laboratory magnetic field breaks the symmetry of the helical ground state, and the superposition of three helical waves in the plane perpendicular to the laboratory field generates a two-dimensional triangular lattice of skyrmions. Noncentrosymmetric helimagnets in B20-type alloys, such as MnSi [2,23], Fe${}_{1-x}$Co${}_{x}$Si [5], and FeGe [3,21] (all of which possess the same cubic chiral space group), have been shown to support subambient temperature skyrmion phases [22]. However, skyrmion formation below room temperature presents an inherent implementation challenge for spintronic applications.

Ref. [33] reported β-Mn type Co-Zn-Mn alloys, specifically Co${}_{8}$Zn${}_{8}$Mn${}_{4}$, to have a triangular lattice thermal equilibrium phase and has since been characterized over a host of its compositional series and a variety of skyrmion phases via Small Angle Neutron Scattering (SANS), Lorentz Transmission Electron Microscopy (LTEM), and magnetization techniques [33,34,35,36,37,38]. Of particular bearing on the materials magnetic properties is the level of Mn-doping, which introduces both magnetic frustration and magnetic disorder due to antiferromagnetically coupled Mn spin correlations [34,37,39] and site mixing of the ferromagnetic Co and antiferromagnetic Mn spins [34,39], respectively. Magnetic anisotropy has also been shown to vary both in magnitude and orientation of its easy axes upon variation of the Co/Mn ratio [36]. It is precisely this cooperative interplay of magnetic anisotropy, spin disorder, and frustration, which stabilizes such a rich energy landscape with a high density of defects in these materials. Accordingly, a myriad of exotic long-period chiral structures/phases have since been realized in the Co-Zn-Mn series, ranging from meron-antimeron lattices generated by in-plane magnetic anisotropy [38] to disconnected low-temperature disordered skyrmion phases stabilized by frustrated interactions [37]. However, the influence of underlying chemical and crystalline disorder on the thermal equilibrium phase remains widely unexplored in these materials. Here, we examine skyrmion ordering protocols in a highly disordered, jammed, thermal equilibrium phase of a polycrystalline sample of Co${}_{8}$Zn${}_{8}$Mn${}_{4}$. Through the application of an ordering technique from [40], we precipitate ordered and oriented skyrmions that yield secondary diffraction rings. We emphasize the influence of anisotropy in Co${}_{8}$Zn${}_{8}$Mn${}_{4}$ and its ability to stabilize the thermal equilibrium skyrmion phase in spite of underlying material disorder. This demonstration of a disordered skyrmion phase in thermal equilibrium contributes to the diverse set of spin textures the Co-Zn-Mn compositional series of skyrmions is able to generate, reinforcing the rich energy landscapes inherent to these materials.

## 2. Synthesis

**Figure 1:**
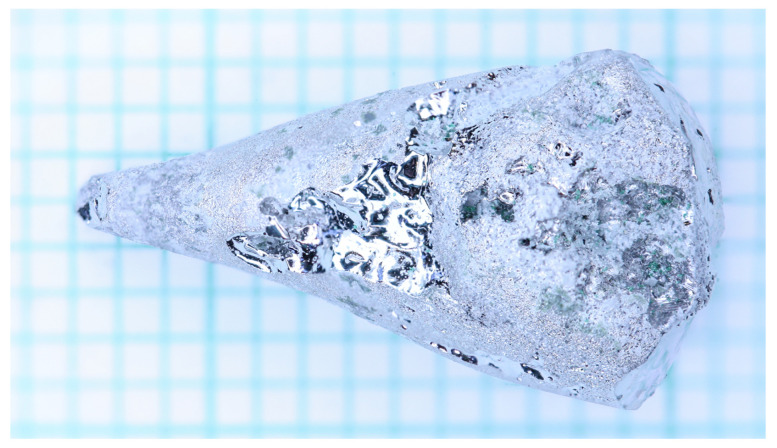
Polycrystalline Co${}_{8}$Zn${}_{8}$Mn${}_{4}$ sample mixed under argon of the dimensions 0.8 cm × 1.4 cm and a mass of 2 g. Each grid line corresponds to 1 mm.

The material was synthesized via the solid state reaction 8Co + 8Zn + 4Mn → Co_8_Zn_8_Mn_4_. The powders were mixed in stoichiometric ratios in an agate mortar under an argon atmosphere. Once thoroughly ground, the resulting mixture was pressed into a pellet, which was then sealed in an evacuated quartz tube with a conically shaped end. The conical shape of the ampoule served to facilitate nucleation along a dominant growth direction, imposed by the geometry of the confining tube. The ampoule was inserted into a furnace at 700 ${}^{\circ }C$, and the temperature was increased to 1025 ${}^{\circ }C$ over the course of 12 h. It was then cooled at a rate of 2 ${}^{\circ }C/h$ until 700 ${}^{\circ }C$ was reached. Finally, it stayed at 700 ${}^{\circ }C$ for 12 h and was removed. The final product was a conical-shaped silver polycrystal (approximately 2–3 grains) with the dimensions 0.8 cm × 1.4 cm (diameter × length) and a mass of 2 g as shown in Figure 1).

**Figure 2:**
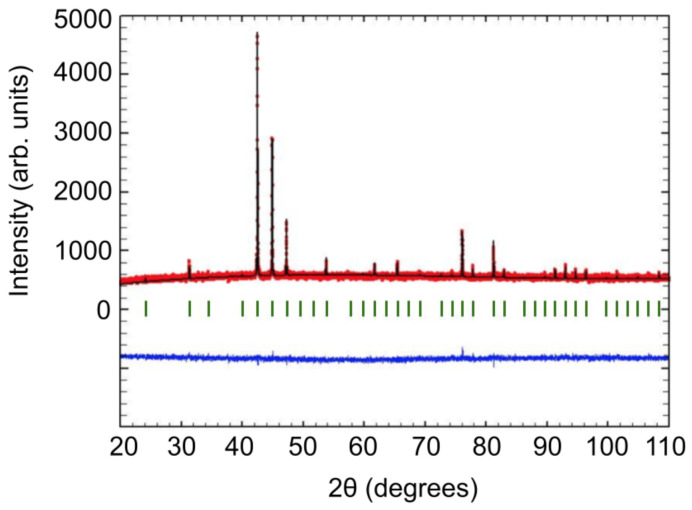
The Rietveld refinement for the powder X-ray diffraction of Co${}_{8}$Zn${}_{8}$Mn${}_{4}$. The black curve is the predicted spectra, the red line is the data, and the blue is the difference between the two. The green vertical lines indicate the locations of the expected peaks. The refinement demonstrates the sample is phase pure with space-group β-Mn and lattice constant 6.37161(1)Å.

The reaction products were analyzed via powder X-ray diffraction in the scattering angular (2θ) range of 20${}^{\circ }$–110${}^{\circ }$ using the Cu $K_{\alpha 1}$ wavelength of 1.54056Å. A Rietveld refinement of the diffraction data to the $P4_{1}32$ space-group (β-Mn-type) was performed using the FullProf program, which we were able to extract a lattice constant of 6.37161(1)Å from. Figure 2 shows the Rietveld refinement for the powder X-ray diffraction spectra, where the red dots are the measured spectra, the black line is the predicted spectra (where the vertical blue lines below indicate expected peak locations), and the blue line is the difference between the two. The sharpness of present peaks (evidenced by the zero slope of the blue curve) and the absence of additional peaks indicate the sample is phase pure.

## 3. Characterization

**Figure 3:**
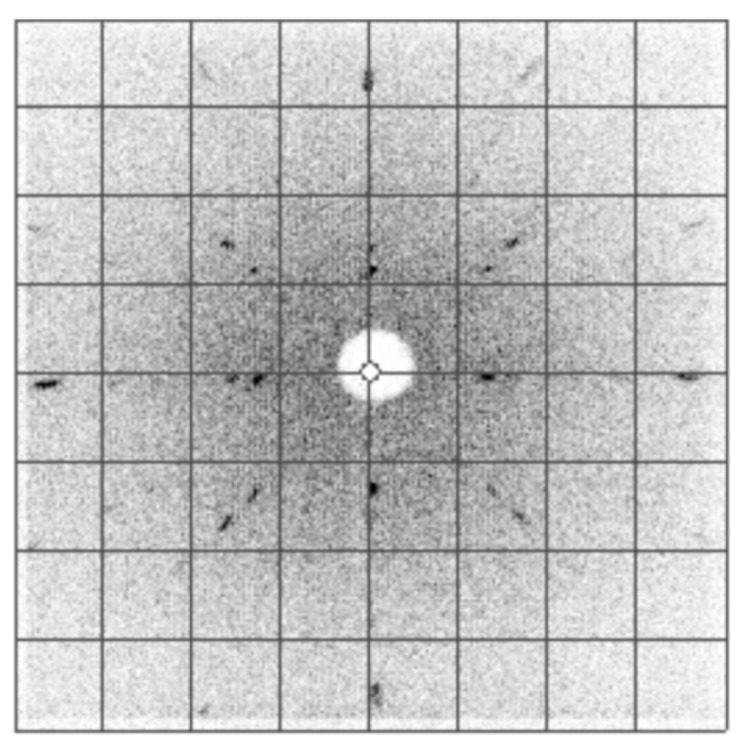
Laue image along (100) direction of the dominant grain on the front face of the cube. All peaks were indexed to the (100) direction, verifying the single grain portion of this material. The four-fold symmetry of the pattern is characteristic for the (100) direction of a cubic crystal.

Backscatter X-ray Laue diffraction was performed as a preliminary investigation of the crystallinity and orientation of the material (Figure 3). Based on a changing diffraction pattern during translation, we were able to identify grain boundaries.

Through systematic scanning and slicing, the polycrystal was cut into a rectangular prism of dimensions 3.4 mm × 3.3 mm × 3.0 mm while mapping the crystal orientation of the polycrystalline sample. The final product was polycrystalline with the (100) direction of the dominant grain along one face of the rectangular prism. Figure 3 shows a Laue pattern for the dominant grain, demonstrating the archetypal four-fold symmetry of the cubic lattice along the (100) direction.

**Figure 4:**
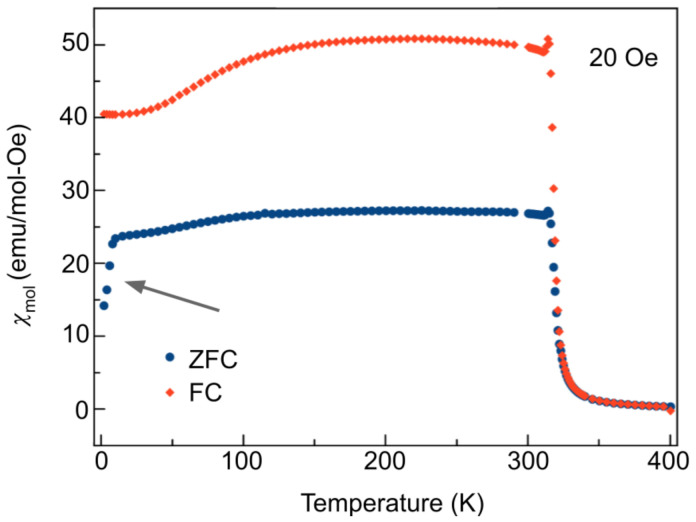
Magnetic susceptibility per mol of Co${}_{8}$Zn${}_{8}$Mn${}_{4}$ after zero-field cooling (ZFC) and field cooling from 400 K in a magnetic field of 20 Oe. The bifurcation behaviour of the ZFC and FC curves at 7 K results from the path-dependent behaviour of the susceptibility (as indicated by the arrow in the ZFC curve), which is indicative of a spin glass transition.

Magnetic susceptibility measurements were performed using a Quantum Design MPMS 5 Superconducting Quantum Interference Device (SQUID) with an AC option installed. The high-temperature ferromagnetic phase was verified via field cooling (FC) from 400 K (Figure 4).

**Figure 5:**
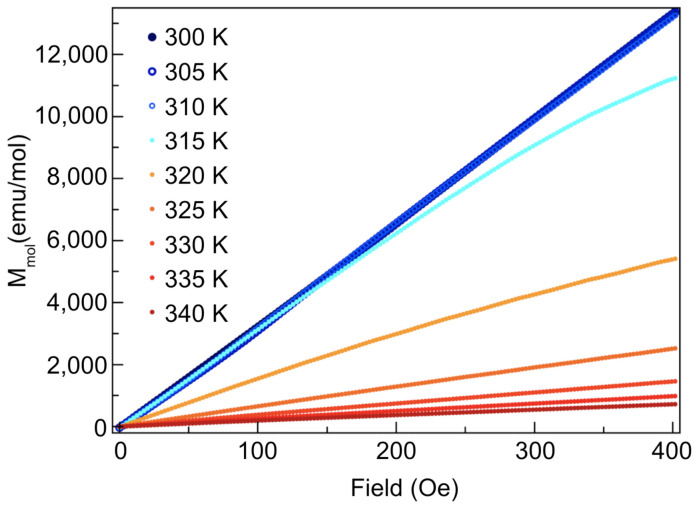
Magnetic field-dependent magnetization upon increasing magnetic fields from 0 Oe to 400 Oe for a temperature range of 300 K–340 K in 5 K increments. Low–high temperature corresponds to blue–red curves. Note a substantial decrease in magnetization for temperatures greater then 320 K, consistent with a paramagnetic phase.

The onset of the transition was found to be 320 K. A Curie–Weiss fit between 350 K and 400 K results in an effective magnetic moment of 1.6 $\mu_{B}$. The high-temperature vertical offset between the FC and ZFC (zero-field cooled) curves is a result of the disparity in magnetization due to the aligned ferromagnetically ordered domains in the FC case, as opposed to the misaligned domains in the ZFC case, which produce a smaller commensurate moment. Further cooling revealed a notable path dependence of the susceptibility on different magnetic field cooling protocols (i.e., ZFC or FC), as is evident by the sharp change in temperature dependence of the ZFC magnetization at around 7 K (illustrated by the arrow in Figure 4). This magnetic behaviour is evidence of a spin glass transition, wherein the marked irreversible magnetic behaviour after field cooling is a result of the cooperative freezing of the spin glass [41]. FC measurements bias the energy landscape, whereas for ZFC measurements, the existence of many metastable states leads to an irreversible path dependence, as the material may not follow the same path to escape the energy valley. The mechanisms underpinning this transition have been previously reported via crystal structure analysis by neutron powder diffraction in [39] as occurring due to site mixing between the Co and Mn atoms on the 8c crystallographic sites, which gives rise to random competition among the ferromagnetic and antiferromagnetic interactions, yielding quenched magnetic disorder. Further susceptibility measurements were carried out to confirm the presence of the skyrmion phase; Figure 5 shows isothermal magnetization measurements as a function of magnetic field for a 20.0 mg polycrystalline piece of the sample. The magnetization measurements were taken while increasing the DC field from 0 Oe to 400 Oe. After, measurements were taken while decreasing the field (not shown). The notable decrease in magnetization for temperatures above 320 K is consistent with exiting an ordered phase into a paramagnetic phase.

**Figure 6:**
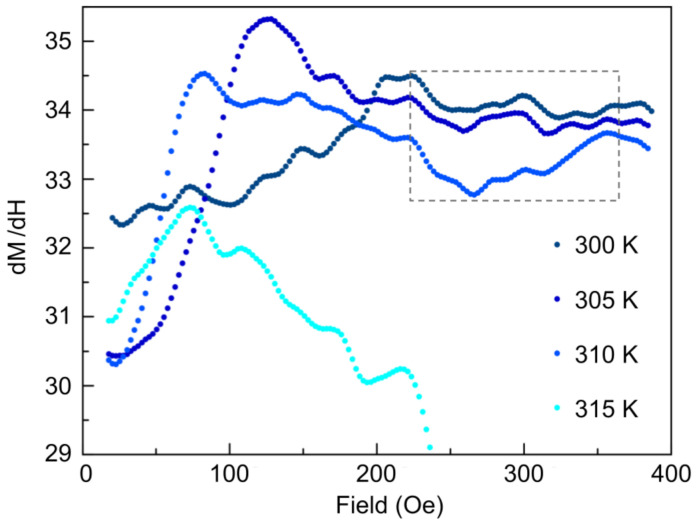
Temperature-dependent isothermal differential magnetic susceptibility upon increasing magnetic fields from 0 Oe to 400 Oe. Low–high temperature corresponds to dark blue–light blue curves. The dip structure (region contained within the rectangular dotted box) is most clearly pronounced for 310 K and presents at a field of ∼200 Oe, indicating the onset of the skyrmion phase.

We performed differential magnetic susceptibility measurements at 300 K, 305 K, 310 K, and 315 K. This was investigated by taking numerical derivatives of the M vs. H curves and, after smoothing the data, show abrupt dips in the susceptibility (with the strongest dip occurring at 310 K contained within the rectangular dotted box in Figure 6), suggestive of a phase transition.

**Figure 7:**
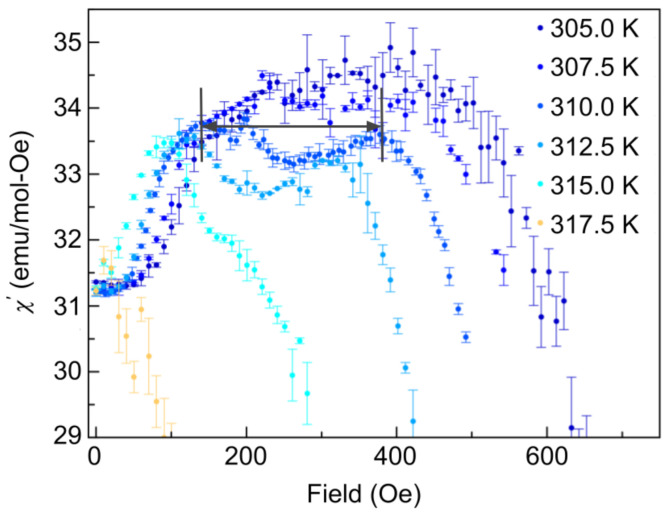
The temperature dependence of AC magnetic susceptibility per mol of Co${}_{8}$Zn${}_{8}$Mn${}_{4}$ over a range of 305 K–317.5 K after increasing magnetic fields from 0 Oe to 500 Oe in a 100 Hz driving field, with an amplitude of 0.1 Oe. Low–high temperature corresponds to dark blue–lighter coloured curves. The skyrmion phase is most pronounced at a temperature of 310 K and is observed to persist in the dip anomaly between 100 Oe and 450 Oe (contained within the region bounded by the lines, as indicated by the arrow). The field value at the minimum of the dip determines the largest and most robust skyrmion phase; these temperature and field parameters are then used for SANS measurements on the material.

AC susceptibility measurements were performed, which depend on $\frac{dM}{dH}$ but do not involve a numerical derivative, which can be susceptible to large fluctuations. AC measurements are, therefore, a much more sensitive technique, yielding a much smaller uncertainty than the above differential magnetic susceptibility measurements. The AC susceptibility measurements show similar peaks as a function of applied field, indicating a phase transition. The most pronounced dip structure is again observed at a temperature of 310 K (contained within the magnetic field range defined by the two lines and arrow in Figure 7), consistent with Figure 6. These dip structures are well-known markers of the temperatures and fields over which the skyrmion phase exists [33]. The AC susceptibility shows much cleaner and more defined dip structures than Figure 6. AC measurements also probe the dynamics of the system and may be included in future work to probe the time scales of the metastable skyrmion phases found below 300 K.

**Figure 8:**
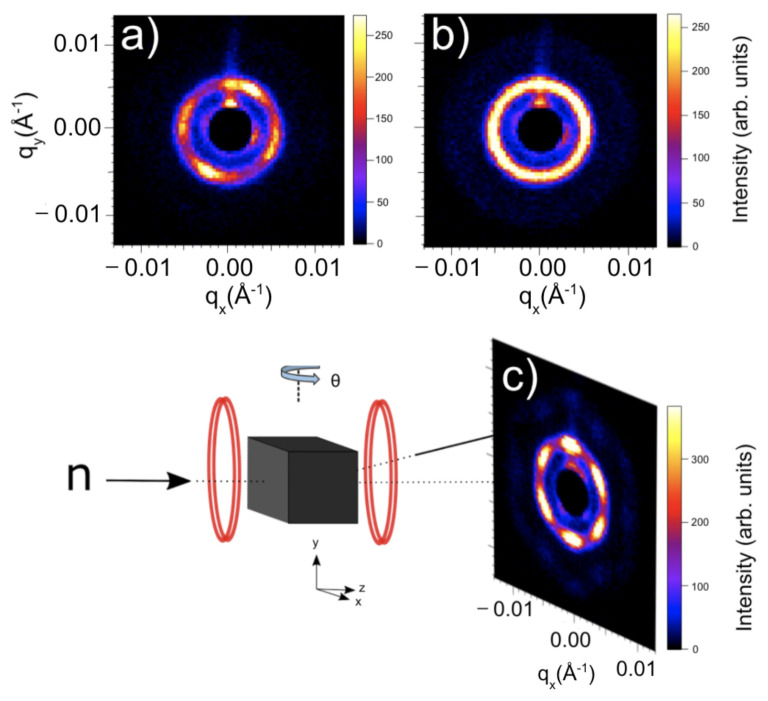
SANS images showing the disordered helical ground state at room temperature in zero field (**a**); the initial scattering ring for disordered skyrmion domains at 310 K in a magnetic field of 250 Oe (**b**); a schematic of the symmetry-breaking field rotation setup; and the SANS image after 30 rotations of the rotation sequence at 310 K in a field of 250 Oe (**c**). The increased intensity/preferential smearing of the peaks in the top right and bottom left diagonals of the four-fold helical image elucidate the anisotropy direction for the crystal. A schematic of the rotation setup illustrates the neutron propagation direction (n) is in the z-direction. For the symmetry-breaking rotation sequence, the sample is rotated symmetrically in the xz plane about θ, with the magnetic field fixed in the z-direction.

We performed unpolarized SANS at the NG7-30m beamline at the National Institute for Standards and Technology (NIST) for a 15 m beam configuration and a neutron wavelength of 6Å [42,43]. At room temperature in zero field, our initial SANS measurements revealed four smeared magnetic satellites atop a circular ring, indicating multi-domain single q-helical structures, with the preferential smearing direction of the peaks elucidating the anisotropy direction (part a) in Figure 8). Upon field cooling through the ferromagnetic phase from 420 K to 310 K, in a field of 250 Oe, a ring developed. The absence of the signature triangular lattice skyrmion hexagonal pattern is a result of the polycrystalline nature of the material; the misalignment of the skyrmion domains breaks the order in many directions, thereby smearing the hexagonal patterns, precipitated by each individual domain, to produce a ring. Using a symmetry-breaking magnetic field sequence [40] where the sample is rotated symmetrically in the static magnetic field to precipitate ordered and oriented skyrmion lattices despite the overwhelming structural disorder, the underlying triangular lattice skyrmion phase was revealed. The development of a first-order ring with six peaks and an additional secondary ring was observed after 10 symmetric rotations. Part c) in Figure 8 shows the fully discernible six-fold primary ring after 30 rotations, accompanied by a second-order ring mimicking the same hexagonal symmetry with 12 peaks. The presence of the secondary ring indicates potential multiple scattering and/or higher order diffraction. The underlying mechanism is left to be investigated for future experiments. The energetics of the skyrmion ordering sequence showed, through micromagnetic simulations, magnetic moments to diverge away from the external field when approaching a magnetic hard axis, consequently increasing the DM energy, resulting in a lattice reorientation [40]. Therefore, the response of our material to the ordering sequence highlights the role of anisotropy in skyrmion formation and reorientation dynamics.

In conclusion, we have successfully demonstrated the synthesis and characterization of the above room temperature bulk disordered triangular lattice skyrmion material Co${}_{8}$Zn${}_{8}$Mn${}_{4}$. Powder X-ray diffraction studies revealed a pure phase, while backscatter Laue diffraction and neutron diffraction indicated a polycrystalline material. SANS measurements demonstrated the underlying rotationally disordered skyrmion domains. The application of the symmetry-breaking rotation sequence [40] precipitated ordered and oriented triangular skyrmion lattices, yielding secondary diffraction rings. These secondary diffraction rings are most likely a combination of double scattering (owing to the thickness of the sample) and higher-order diffraction, which, in turn, elucidates the effectiveness of the technique in [40] for ordering and even promoting the growth of skyrmions despite the presence of disorder, thereby producing long-range order. While the phenomena of skyrmion ordering is by no means novel, experimental demonstrations of the conversion from disordered chiral states to ordered skyrmion lattice forms—in varying host compounds—contributes fundamental insight into the nature of skyrmion formation energetics, pinning phenomena, and stabilization mechanisms. This study serves as a unique demonstration of the interplay of anisotropy and disorder in the thermal equilibrium phase for the Co-Zn-Mn skyrmion series, reinforcing the influence of crystalline disorder and material defects on skyrmion formation and orientations in skyrmion phases stabilized by thermal fluctuations. Future experiments may explore the ratio of multiple scattering to higher-order diffraction through the use of Renninger scans [44]. Furthermore, we intend to use a newly developed reconstruction algorithm [45] to perform 3D tomography of skyrmion topological transitions in the bulk, as well as incorporate spin components to explore the structure of the neutron wavefunction after passing through a skyrmion sample [46,47,48].

## Data Availability

Data available upon request.

## Abbreviations

The following abbreviations are used in this manuscript:

MDPI	Multidisciplinary Digital Publishing Institute
DOAJ	Directory of open access journals
TLA	Three letter acronym
LD	Linear dichroism
